# Potential Adverse Effects of Violent Video Gaming: Interpersonal- Affective Traits Are Rather Impaired Than Disinhibition in Young Adults

**DOI:** 10.3389/fpsyg.2018.00736

**Published:** 2018-05-16

**Authors:** Ann-Christin S. Kimmig, Gerda Andringa, Birgit Derntl

**Affiliations:** ^1^Innovative Neuroimaging, Department of Psychiatry and Psychotherapy, University of Tübingen, Tübingen, Germany; ^2^International Max Planck Research School for Cognitive and Systems Neuroscience, University of Tübingen, Tübingen, Germany; ^3^Department of Science, University College Roosevelt, Middelburg, Netherlands; ^4^Werner Reichardt Center for Integrative Neuroscience, University of Tübingen, Tübingen, Germany; ^5^LEAD Graduate School and Research Network, University of Tübingen, Tübingen, Germany

**Keywords:** violent video gaming, antisocial traits, adverse effects, disinhibition, interpersonal-affective deficits

## Abstract

The increasing trend of mass shootings, which were associated with excessive use of violent video games, fueled the debate of possible effects violent video games may have on adolescents and young adults. The aim of this study was to investigate the possible link between violent video gaming effects and the disposition of adverse behavior traits such as interpersonal-affective deficits and disinhibition. Data of 167 young adults, collected by an online questionnaire battery, were analyzed for lifetime video game exposure differences (i.e., non-gamers, non-violent video gamers, stopped violent video game users, and ongoing violent video game users) as well as for recent exposure effects on adverse behavior traits (Levenson’s Psychopathy Scale), while controlling for other potentially confounding lifestyle factors. While interpersonal-affective deficits were significantly higher in participants with ongoing violent video game exposure compared to non-gamers and non-violent video gamers, disinhibition was significantly higher in both – stopped and ongoing – violent video game exposure groups compared to non-gamers. Recent violent video game exposure was a stronger predictor for interpersonal-affective deficits, but was also significant for disinhibition. Considering that we observed small to medium effects in a sample of young adults with little to moderate use of violent video games highlights the importance of further investigating the potential adverse effects of violent video games on quality of social relationships.

## Introduction

Video game exposure in adolescents aged between 8 and 18 years has been estimated to be around 15 h per week in the United States, with boys playing more than girls (Gentile, 2009). Furthermore, the considerable growth of the video game industry over the past decades implicates an increase in the exposure to video games. Thus, it is crucial to investigate possible effects on users. Video gaming is associated with beneficial effects such as improved cognitive functions, e.g., quick decision-making (Green et al., 2010; Bavelier et al., 2012), cognitive flexibility (Colzato et al., 2010), and increased attentional resources (Green and Bavelier, 2003; Bavelier et al., 2012; Vallett et al., 2013); however, it may also be a risk factor in terms of promoting aggressive behavior (Anderson, 2004; Anderson et al., 2010).

The concerns about potential adverse effects of video games can predominantly be attributed to violent video games. Even though numerous studies support the claim that violent video games affect adolescents’ attitudes and behaviors negatively (Anderson, 2004; Fraser et al., 2012; Saleem et al., 2012; DeLisi et al., 2013; Gabbiadini et al., 2013; Siyez and Baran, 2017), there are also studies in which no effects are found (Adachi and Willoughby, 2011; Parkes et al., 2013; Szycik et al., 2017; study suggesting publication bias: Ferguson, 2007). Therefore, the debate on potential beneficial vs. adverse effects from violent video gaming remains heated.

Apart from an overall relationship between gaming and negative behavior, research suggests a link between violent video game exposure and isolated behavioral traits and behaviors, such as empathy, morality, and aggression. The analysis of longitudinal, experimental and cross-sectional studies suggests that there is a negative relationship between violent video gaming and empathy (Funk et al., 2004; Anderson et al., 2010; Fraser et al., 2012). Moreover decreased practice of empathy-related concepts (Klimmt et al., 2006), such as sympathy and perspective taking, are believed to be also implicated in the delay of moral development found in adolescents with high violent video game exposure (Bajovic, 2013). These adolescents seem to exhibit not only immature moral reasoning but also moral disengagement (Hartmann and Vorderer, 2010; Gabbiadini et al., 2012, 2013). Furthermore, numerous studies with different methodological approaches – including short-term correlational (Gentile et al., 2004; Wei, 2007; DeLisi et al., 2013), experimental studies (Anderson and Dill, 2000; Saleem et al., 2012; Gabbiadini et al., 2013), longitudinal studies (Wallenius and Punamäki, 2008; Möller and Krahé, 2009; Willoughby et al., 2012), and meta-analyses (Anderson and Bushman, 2001; Anderson, 2004; Anderson et al., 2010; Greitemeyer and Mügge, 2014) – provide strong evidence for a link between violent video game exposure and aggression. For example, the well-controlled longitudinal study by Willoughby et al. (2012) indicated a causal effect of violent video game exposure on aggressive affect, attitudes, and behavior in a large sample of adolescents.

While, most studies focused on the effect of violent games on a single trait or behavior – such as empathy, morality or aggression (Anderson et al., 2010; Willoughby et al., 2012; Gabbiadini et al., 2013) – adverse behavior usually arises from a combination of traits. These can be grouped into two domains: *interpersonal-affective deficits* including social efficacy, pathological lying, and manipulative behavior and *disinhibition* including lack of impulse control, lack of planning, irresponsibility, and immediate reward seeking (Hare, 2003). The interplay, as well as the distinct contributions, of these two domains is typically used to assess the degree of psychopathy and more importantly are thought to give rise to antisocial behavior including aggression, delinquency, and immoral decisions (Cima et al., 2010; Seara-Cardoso et al., 2012; DeLisi et al., 2013).

The importance to investigate a combination of certain traits related to expression of adverse behavior is highlighted by DeLisi et al.’s (2013) findings: in their study, violent video game exposure was a significant risk factor for adverse behavior (i.e., delinquency/violence) in a sample of institutionalized juvenile delinquents. The strongest predictor for delinquency and violence were, however, pre-existing deficits in inhibition and interpersonal-affective abilities. Unfortunately, the interaction between violent video game use and psychopathic trait disposition was not directly analyzed. However, DeLisi et al. (2013) suggest that especially individuals with pre-existing deficits in disinhibition and interpersonal-affective abilities may be affected more by violent video game use resulting in a higher likelihood of the expression of adverse behavior.

Considering the increasing trend of extensive violent video games use (Gentile, 2009), however, it is crucial to investigate whether psychopathic trait disposition is also influenced in violent video game consumers without a history of delinquency. This study aimed to shed light on the possible link of violent video game exposure and the expression of traits usually contributing to adverse behavior including interpersonal-affective and disinhibition traits. The potential relationships were explored empirically using the Levenson’s Psychopathy Self-Report Scale (Levenson et al., 1995) in a young adult population to measure the aforementioned traits. Unlike in many previous studies, this gender-mixed sample contained a significantly higher proportion of females. Furthermore, the video game consumption of this group can be classified as low to medium. Therefore, this study does not only allow to gain some more insight into video gaming also in female users, as most studies include mainly males, but also on the influences of low to moderate exposure on a young adult group as mostly potential effects of excessive use are studied. Moreover, insight into the potential reversibility of adverse effects induced by violent video game exposure was studied by comparing subjects with a history of life-time violent video game exposure to subjects with ongoing exposure. Next to the unconventional approach of comparing ongoing to stopped violent video game exposure in such a way, we decided not only to investigate potential links to lifetime exposure, but also additionally to examine the predictive power of recent (violent) video game exposure on interpersonal-affective and disinhibition trait expression while controlling for some possible lifestyle-related confounders. This approach was chosen to explore recent exposure effects more closely.

## Materials and Methods

### Participants

The data from a total of 203 participants was collected using an online-questionnaire battery shared via social media. Inclusion criteria included: age between 16 and 24 years and good knowledge of the English language. Several participants had to be excluded due to age inclusion criteria (*n* = 21) and missing data on the items regarding gender (*n* = 3), mental health (*n* = 6), and drug use in the past 2 weeks (*n* = 6). Hence, data of 167 individuals was included in the analysis. Considering the video gaming behavior in this sample, four main groups were identified, namely non-video game users (*NVG*, *n* = 38), non-violent video game users (*NVVG*, *n* = 52), video-game users who stopped playing violent games more than 2 months ago (*SVVG*, *n* = 39), and violent video game users (*VVG*, *n* = 38). These groups were formed according to self-reported exposure to video games in the past. Participants themselves indicated whether they used violent or non-violent games. This distinction was furthermore tested by having experts rating the most commonly named games on the content of violence. Lastly, it is noteworthy that not only first-person shooter games were labeled as violent, but also strategy games which include virtual violence.

#### Sample Characteristics

The majority of participants were female (65%) and aged between 19 and 21 years (70%). Furthermore, 90% of the sample was enrolled at a university [mostly at University College Roosevelt (UCR) – an honor’s college of Utrecht University located in the South West of Netherlands]. The remaining participants either studied at a lower level of higher education or were still at high school. Participants were fairly evenly distributed over a variety of educational fields. An overview of demographics, video game behavior, and psychopathic trait expression as well as lifestyle factors for all four groups can be found below in **Table 1**.

**Table 1:** General sample characteristics.

Video game groups	*NVG^∗^*	*NVVG^∗^*	*SVVG^∗^*	*VVG^∗^*
***N***	38	52	39	38
Gender (male/female)	4/34	6/46	23/16	25/13
Age (n)				
16 to 18	1	4	6	11
19 to 21	29	43	27	18
22 to 24	8	5	6	9
Educational level (*n*)				
High school	0	1	4	1
Vocational school	1	4	1	4
University	37	47	34	33
Psychological problems (yes/no)	5/33	14/38	6/33	10/28

**Media exposure**	**Median**	**Median**	**Median**	**Median**

**Lifetime exposure**				
Video game use (years)	–	4–7	8–11	8–11
Video game intensity (times/per month)	–	Couple of times/month	1–2/week	1–2/week
**Recent exposure (past week)**				
Non-violent video games (h)	–	<1	<1	<1
Violent video games (h)	–	–	–	1–3
Violent movies (frequency)	0	<1–2	0	1–2

**Levenson’s Psychopathy Scale**	**Mean (SD)**	**Mean (SD)**	**Mean (SD)**	**Mean (SD)**

Interpersonal-affective deficits	30.13 (8.75)	30.98 (7.90)	37.26 (10.03)	39.26 (14.03)
Disinhibition	20.24 (6.29)	23.42 (5.66)	23.62 (5.62)	22.74 (5.43)
Overall psychopathy score	50.37 (11.32)	54.40 (11.55)	60.87 (12.68)	62.00 (16.32)

**Lifestyle factors**				

Sleep (h)	6–8	6–8	6–8	6–8
Stress (averaged total, range: 1–4)	2.53 (0.48)	2.71 (0.38)	2.65 (0.49)	2.24 (0.73)
Physical exercise (times/week)	1–2	1–2	1–2	1–2
Smoking (no. of days/month)	0 (0 – >20)	0 (0 – >20)	0 (0 – >20)	0 (0 – >20)
Alcohol consumption (no. of days/month)	2	2	2	2
Drug use past 2 weeks (yes/no)	2/36	6/46	2/37	4/34

*^∗^NVG, no regular video game use; NVVG, non-violent video game use only; SVVG, stopped violent video game use; VVG, ongoing violent video game use.*

### Measures

The survey software *Limesurvey* was used to collect the data online. The questionnaire, presented in English language, contained six subsections each collecting distinct information (namely, general information, video game behavior, other lifestyle factors, and interpersonal-affective as well as disinhibition traits) necessary for this study. In total, this questionnaire required approximately 15–20 min for completion.

#### Demographics

The first section was concerned with the participant’s gender, age, and educational level (high school, vocational school, university). Multiple choice questions were used to ask for gender and age, whereas text boxes were provided for answers regarding educational level and field of study in order to avoid restricting answer possibilities.

#### Video Gaming Behavior

The second part (6 items) of the questionnaire retrieved information about the individual’s gaming behavior. Questions concerning duration [in years, from 0 (none) to 5 ( > 11 years)] and intensity [frequency, 0 (less than once a month) to 5 (at least once a day)] of video game use as well as recent video game exposure within the past week [h/week, from 0 (less than 1 h) to 4 (>10 h)] were either adapted from a previous studies, templates available on *Limesurvey* or have been designed to tap into information specific for this study.

#### Other Important Lifestyle Factors

In this section, information about possible confounding factors was measured. Three of the questions about stress were taken from the Overload questionnaire using 4-point Likert scale originally developed by Everly (Doyle et al., 2014). The four items referring to alcohol and tobacco use [in days/months (0 (none) – 6 (every day) or in number/day (cigarettes: 0 (<1) to 5 (>20)] were inserted from the *Youth Risk Behavior Survey* (Centers for Disease Control and Prevention, 2013). In order to control for other possible confounding variables, four additional questions regarding drug use in the past 2 weeks (yes/no), mental health (psychological problems yes/no), physical exercise [frequency/week, rating scale from 0 (none) to 4 (>7 times)] and exposure to violent movies in the past week [frequency, rating scale from 0 (none) to 4 (>7 times)] were included.

#### Interpersonal-Affective and Disinhibition Traits

The Levenson’s Psychopathy Scale (Levenson et al., 1995) was used to measure adverse traits. The 26 items of this questionnaire can be divided up into two scales referring to primary psychopathy – deficits in interpersonal and affective processing – (16 items; possible scores: 16–80) and secondary psychopathic traits (10 items; possible scores: 10–50), which are characterized by a defiant lifestyle with antisocial tendencies due to problems with disinhibition. The Levenson’s Psychopathy Scale was chosen for this research project because it has been validated in non-institutionalized, healthy populations, especially students (Levenson et al., 1995; Lynam et al., 1999). Furthermore, the relative shortness of the questionnaire is advantageous as it keeps participants motivated to complete it.

### Procedure

The data was collected via social media platforms (e.g., Facebook). Participants were told that the aim of this study was to investigate the possible relationship between certain lifestyle factors and personal attitudes or beliefs toward the self and others. This study was approved by the internal ethics committee of the University College Roosevelt. Before starting the questionnaire, all participants gave informed consent. They were informed that they could stop answering questions at any point without any justification.

### Data Analysis

The statistical analyses of the collected data were carried out using IBM SPSS 24. ANCOVA tests were used to investigate whether the psychopathy scores^1^ differed among the various video game use groups when controlling for possible confounding effects including gender, age, educational level, drug use, psychological problems, physical exercise, and violent movie exposure. Due to violating the assumption of independence between covariates and group effects, violent movie exposure, and duration of videogame exposure in years were not included in the main analysis as common underlying mechanism can be expected between these covariates and the VG group factor. Multiple ANCOVA analyses were used instead of a MANCOVA because of the explorative nature of this analysis and the violation of multivariate normality (Huberty and Morris, 1989). In order to investigate group differences more precisely, planned contrasts were tested. Simple contrasts, with the non-gamers as reference group, were chosen as this allowed not only differentiating between potential effects of violent video gaming, but also for non-violent video gaming. First, the differences between groups of the total psychopathy traits were analyzed. Subsequently, the sub-scores for interpersonal-affective deficits and disinhibition were examined to disentangle contributions to group differences of these different domains of traits. Pearson’s correlations were used to assess the direction of found relationships.

To test the relation between short-term exposure (i.e., exposure in the past week) to violent video gaming and trait expressions, three multiple regression models with interpersonal-affective deficits, disinhibition, and the total collections of traits as dependent variables, were built. After adding the video gaming variables in the first two blocks, the other potential confounding variables were added in additional blocks to see how the relationship changed for violent video game exposure and psychopathic traits when controlling for these factors. Non-violent video game exposure was entered prior to violent video game exposure to investigate whether there is an added and distinct influence of recent violent video game exposure in particular.

## Results

### Lifetime Video-Game Use and Psychopathy Scores

Possible connections to long-term video game use and trait expression were tested by multiple ANCOVAs. **Table 2** summarizes the meaningful differences found in the psychopathy-related trait scores for the different video game exposure groups and other potential confounders.

**Table 2:** Overview of significant results from ANCOVA analyses.

	*F*	df	*p-value*	*partial η*^2^
**Overall trait expression**				
Smoking	7.07	1	0.01	0.04
*Video game exposure*	3.95	3	0.01	0.07
**Interpersonal-affective deficits**				
Alcohol consumption	4.53	1	0.04	0.03
*Video game exposure*	3.12	3	0.03	0.06
**Disinhibition**				
Stress	4.41	1	0.04	0.03
Alcohol consumption	5.04	1	0.03	0.03
Smoking	13.21	1	<0.01	0.08
*Video game exposure*	3.26	3	0.02	0.06

Due to the violation of the assumption regarding independence of covariate and treatment effects, the total years of exposure to video games was not included in the analysis presented below. There was a significant difference in years of exposure between the NVG group and all video gaming groups (all |*mean difference*|≥ 3.33, all |*p*|≤ 0.01) mainly leading to the violation of the aforementioned assumption. To check whether duration of video game exposure explains a substantial part of variance in trait expression, a separate analysis was run with years of video game exposure as group variable [i.e., no exposure, medium duration (>1–7 years) and long-term exposure (>8 years)] instead of the video game type groups. In this additional analysis, duration of video game use was not a significant factor for interpersonal-affective deficits [*F*(2,154) = 1.25, *p* = 0.29, *partial η^2^* = 0.02]. However, for disinhibition [*F*(2,154) = 4.60, *p* = 0.01, *partial η^2^* = 0.06] and overall trait expression [*F*(2,154) = 3.17, *p* = 0.05, *partial η^2^* = 0.04], the duration of video game exposure was a significant factor. *Post hoc* analyses of these effects indicated that only participants, who have never been exposed to video games, scored significantly lower [all |*mean difference*|≥ 3.06, all *p* (one-tailed) < 0.02], whereas there were no differences in medium to long-term exposure groups (all *p* > 0.05). Therefore, the observed effect may not necessarily be explained by varying degrees of exposure duration, but more likely by the previous use or non-use of video games, which was already conceptualized in the video game groups in the main analyses below.

#### Overall Trait Expression and Lifetime Exposure

To begin with, a potential effect of video gaming behavior on the overall trait expression score was investigated. The only covariate, which turned out to be significantly related to the total trait expression score, was smoking, *F*(1,154) = 7.07, *p* < 0.01, *r* = 0.50. All remaining covariates did not have a meaningful relationship with the overall trait expression (|all *F*|≤ 1.95, all *p* < 0.17).

**FIGURE 1:**
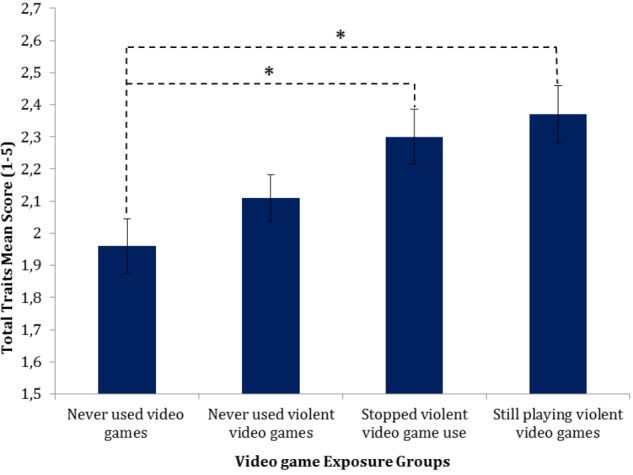
Normalized means plot of overall trait expression across video game groups. Error bars denote standard errors (SEs). *^∗^*Sidak corrected *p*-value < 0.05.

A significant overall effect on total trait expression was found for the video gaming groups, *F*(3,154) = 3.95, *p* = 0.01, *partial η^2^* = 0.07. When examining the differences between the individual video gaming groups via planned simple contrasts, there was no significant difference between NVG and NVVG, *t*(154) = 1.40, *p* = 0.16, *r* = 0.11. However, a significant increase in the total trait expression from NVG to SVVG exposure, *t*(154) = 2.73, *p* < 0.01, *r* = 0.22, and to VVG exposure, *t*(154) = 3.18, *p* < 0.01, *r* = 0.25, was detected. The means plot in **Figure 1** shows the trend of the overall trait expression means across the different groups when controlling for possible confounding variables. Even though total trait scores are significantly higher in groups which have been exposed to violent video games in the past, it is important to point out that the trait expression is still on a lower to midrange level (1 = no tendency, 5 = strong tendency).

#### Interpersonal-Affective Deficits and Lifetime Exposure

When examining the association between video game use with interpersonal-affective deficits specifically while controlling for potential confounders, only the covariate alcohol consumption over the past months, *F*(1,154) = 4.53, *p* = 0.04, *r* = 0.17, turned out significant. The positive correlation (Pearson’s *r* = 0.20, *p* < 0.01) suggests that increased alcohol use is therefore linked to a higher interpersonal-affective deficits score. All other covariates remained insignificant (|all *F*|≤ 2.38, all *p* > 0.10).

**FIGURE 2:**
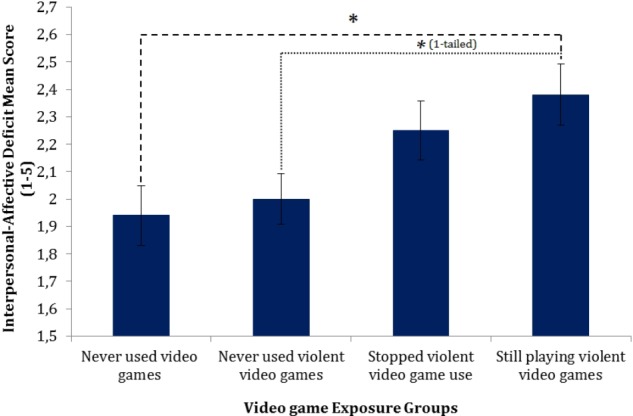
Normalized means plot of interpersonal-affective deficits across video game groups. Error bars denote SEs. *^∗^*Sidak corrected *p*-value < 0.05.

There was also a significant effect of video gaming behavior, while controlling for potential confounders, on interpersonal-affective deficits, *F*(3,154) = 3.12, *p* = 0.03, *partial η^2^* = 0.06. Planned contrasts were used to determine differences between groups. There was no distinction between NVG exposure and NVVG exposure, *t*(154) = 0.44, *p* = 0.67, *r* = 0.04. The differences between NVG exposure and SVVG exposure more than 2 months ago, however, just failed to reach significance, *t*(154) = 1.96, *p* = 0.05, *r* = 0.16. The comparison between no exposure vs. ongoing violent video game exposure, on the other hand, surpassed the statistical threshold indicating greater interpersonal-affective deficits in the VVG group compared to NVG participants, *t*(154) = 2.71, *p* = 0.01, *r* = 0.21. The trend of interpersonal-affective deficit scores across these different groups can be viewed in **Figure 2**. Yet again, even the highest means are in the lower to midrange level of trait expression scores.

#### Disinhibition and Lifetime Exposure

In this statistical analysis, several covariates were significantly related to disinhibition scores. First, stress was positively related (Pearson’s *r* = 0.23, *p* < 0.01) to disinhibition scores, *F*(1,154) = 4.41, *p* = 0.04, *r* = 0.34, and so was smoking (Pearson’s *r* = 0.25, *p* < 0.01), *F*(1,154) = 13.21, *p* < 0.01, *r* = 0.73. Alcohol consumption, on the other hand, was negatively associated (*Pearson’s r* = -0.07, *p* > 0.05) with disinhibition scores, *F*(1,154) = 5.04, *p* = 0.03, *r* = 0.38. This indicates that higher levels of perceived stress and smoking are related to more problems in cognitive control/disinhibition, whereas more frequent alcohol consumption was linked to less disinhibition in this student sample. Notably, the negative correlation with alcohol consumption is very small and insignificant. All the other covariates were insignificant (|all F|≤ 0.78, all *p* > 0.39).

**FIGURE 3:**
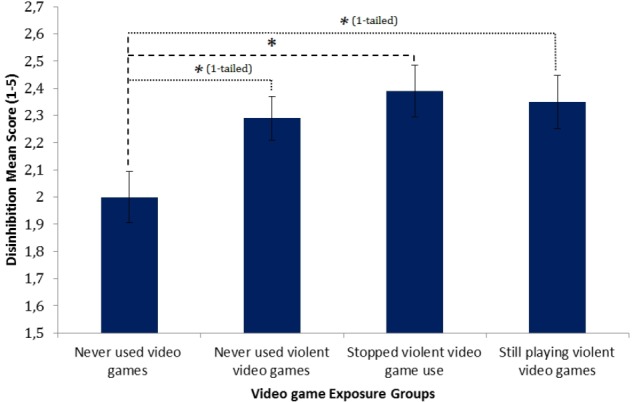
Normalized means plot of disinhibition across video game groups. Error bars denote SEs. *^∗^*Sidak corrected *p*-value < 0.05.

When controlling for all possible confounders, video gaming still had a significant effect on the disinhibition score, *F*(3,154) = 3.26, *p* = 0.02, *partial η^2^* = 0.06. Planned simple contrasts with the non-gamers as reference group revealed that exposure to any video games was significantly related to higher disinhibition traits, as there was a meaningful difference to the NVG group, *t*(154) = 2.47, *p* = 0.02, *r* = 0.20, the SVVG group, *t*(154) = 2.78, *p* < 0.01, *r* = 0.22, and VVG group, *t*(154) = 2.46, *p* = 0.02, *r* = 0.19. **Figure 3** displays the trend of secondary psychopathy scores across the different video game groups when controlling for the covariates.

Even though mean scores are significantly higher, all means remain in the range of low to medium levels of secondary psychopathy. This is also reflected by the rather small effect sizes (*r*), which range from 0.19 to 0.22.

### Recent (Violent) Video Game Exposure: Multiple Regressions of Trait Expression Scores

Three multiple regressions – each with a different trait expression score – have been built to investigate the short-term link (i.e., considering the past week only) of violent video game exposure with the different psychopathic trait domains. **Table 3** provides an overview of the three final multiple regression models.

**Table 3:** Multiple regressions on different trait expression scores.

	*Overall Traits*			*Interpersonal-affective deficits*			*Disinhibition*		
Final Model	*B*	*SE B*	*β*	*B*	*SE B*	*β*	*B*	*SE B*	*β*
Constant	37.68	17.48	-	21.15	13.64	-	16.53	7.74	-
Non-violent video games	0.85	1.26	0.05	0.63	0.98	0.05	0.22	0.56	0.03
Violent video games	3.49	1.26	0.23**	2.49	0.99	0.21*	1.00	0.56	0.16*
Gender	4.22	2.37	0.15	4.53	1.85	0.20*	-0.31	1.05	-0.03
Age	-3.02	1.94	-0.12	-1.87	1.52	-0.09	-1.15	0.86	-0.11
Educational level	-0.93	2.47	-0.03	-1.11	1.92	-0.04	0.17	1.09	0.01
Stress	3.53	2.19	0.14	0.80	1.71	0.04	2.72	0.97	0.25**
Sleep	-0.66	2.24	-0.02	-0.32	1.84	-0.01	-0.34	1.04	-0.03
Psychological problems	3.94	2.57	0.12	2.88	2.00	0.11	1.05	1.14	0.07
Physical exercise	-1.32	1.06	-0.09	-1.16	0.83	-0.10	-0.17	0.47	-0.03
Violent movies	2.54	1.15	0.17*	2.08	0.90	0.18*	0.47	0.51	0.08
Alcohol consumption	0.45	0.74	0.05	1.14	0.58	0.16	-0.64	0.33	-0.18*
Smoking	1.33	0.68	0.16*	0.41	0.53	0.06	0.92	0.30	0.26**
Drug use	2.11	3.87	0.04	2.47	3.02	0.06	-0.36	1.71	-0.02

*^∗^For overall traits R^2^ = 0.02 for Model 1, ΔR^2^ = 0.07^∗∗^ for Model 2, ΔR^2^ = 0.03 for Model 3, ΔR^2^ = 0.11^∗∗^ for Model 4. For interpersonal-affective deficits R^2^ = 0.02 for Model 1, ΔR^2^ = 0.08^∗∗^ for Model 2, ΔR^2^ = 0.05^∗^ for Model 3, ΔR^2^ = 0.09^∗^ for Model 4. For disinhibition R^2^ = 0.00 for Model 1, ΔR^2^ = 0.01 for Model 2, ΔR^2^ = 0.01 for Model 3, ΔR^2^ = 0.14^∗∗^ for Model 4. ^∗^p < 0.05, ^∗∗^p < 0.01.*

#### Overall Trait Expression and Recent Exposure

Before examining the trait sub-scores separately, it is interesting to analyze whether short-term exposure to violent video games is linked to the overall trait expression score.

Overall, the final model explains 22% of the variance in total scores (*R*^2^ = 0.22, *F*(13,153) = 3.30, *p* < 0.01). The addition of violent video game exposure to the first block led to a significant improvement in model fit [Δ*R*^2^ = 0.07, Δ*F*(1,164) = 12.93, *p* < 0.01] and indicates that violent video game exposure explains about 7% of the variance in overall trait expression scores. The inclusion of gender, age, and educational level failed to significantly improved the model [Δ*R*^2^ = 0.03, Δ*F*(3,161) = 3.40, *p* = 0.20]. Nevertheless, the last block led to a significantly better model fit [Δ*R*^2^ = 0.11, Δ*F*(8,153) = 2.31, *p* = 0.01].

In the final model, violent video game exposure (β = 0.23, *p* < 0.01), violent movie exposure (β = 0.17, *p* = 0.03) and smoking (β = 0.16, *p* = 0.05) were significant predictors of total trait expression. The B-values indicate that for each unit of smoking [range: 0 (none)–6 (every day)] an increase of 1.3 points on the overall trait scale is expected. For violent movie exposure a rise of about 2.5 points is predicted for each unit increase – amounting to a total difference of 10 points (about 9.6%) between no recent exposure and more than 7 times per week. Furthermore, the B-value of 3.49 in the final model indicates that increasing one level in violent video game exposure leads to an increase of approximately 3.5 points on the overall trait scale. This amounts to an increase of 16.8% (17.5 points) from no exposure in the past week to 10 or more hours of violent video game exposure.

The effect size, β, for violent video game exposure decreases from 0.27 in the second model to 0.23 in the final model. This decrease is likely due to some shared variance with other predictors such as gender and violent movie exposure.

#### Interpersonal-Affective Deficits and Recent Exposure

The addition of violent video game exposure to the first model [*R*^2^ = 0.02, *F*(1,165) = 3.27, *p* = 0.07] resulted in a significant improvement of the model fit [Δ*R*^2^ = 0.08, Δ*F*(1,164) = 15.41, *p* < 0.01]. However, also the addition of the demographic variables in the third model [Δ*R*^2^ = 0.05, Δ*F*(3,161) = 3.40, *p* = 0.02] and the inclusion of other factors, which may confound the violent video game relationship to interpersonal-affective traits improved the model fit significantly [Δ*R*^2^ = 0.09, Δ*F*(8,156) = 2.31, *p* = 0.02]. The final model in **Table 3** explains approximately 25% of the variance in the interpersonal-affective trait scores [*R*^2^ = 0.25, *F*(13,153) = 3.88, *p* < 0.01].

Violent video gaming (β = 0.21, *p* = 0.01), violent movies (β = 0.18, *p* = 0.02), and gender (β = 0.20, *p* = 0.02) were found to significantly predict a change in interpersonal-affective trait scores. Alcohol consumption was on the verge of being a significant predictor for interpersonal-affective traits (β = 0.16, *p* = 0.05).

Since violent video game exposure is the main interest in this analysis, the change of its β value over the different models is examined more closely. When adding violent video game exposure to the regression, there was a significant increase in model fit, which indicates that violent video game exposure explains about 8% of the interpersonal-affective trait score variance. In the third model then there was a quite large drop of the β value of the violent video game exposure variable from 0.29 to 0.21. This drop could be explained by the shared variance between violent video game exposure and gender. The β then remains the same in the final model (β = 0.21). Even though the unique effect of violent video game exposure drops across the different models, its effect remains significant and relatively large compared to the other factors. The B-value of 2.49 in the final model indicates that increasing one level in violent video game exposure leads to an increase of approximately 2.5 points on the interpersonal-affective deficit scale. This amounts to an increase of 19.5% (12.5 points) from no exposure in the past week to 10 or more hours of violent video game exposure.

Considering the other significant predictors, they have similar distinct effects with β values of 0.18 (violent movies) to 0.20 (gender). The B-values suggest that being male is associated with an increase in the interpersonal-affective trait score by 4.5 points and increasing one unit of violent movie exposure is related to a rise of 2.1 points.

#### Disinhibition and Recent Exposure

The final model for disinhibition traits has a worse model fit than the interpersonal-affective traits model, as it only explains 16% of the variance [*R*^2^ = 0.16, *F*(13,153) = 2.29, *p* < 0.01]. The video game exposure predictors were not significant in explaining the variation of disinhibition scores, as they only predict 1% of the variance [*R*^2^ = 0.01, *F*(2,164) = 0.77, *p* = 0.47]. Furthermore, the demographic variables gender, age, and education did also not improve the model fit significantly [Δ*R*^2^ = 0.01, Δ*F*(3,161) = 0.72, *p* = 0.54]. However, adding the last block with the remaining predictors, such as violent movie exposure, stress, physical exercise and drug use, did significantly improve the model fit [Δ*R*^2^ = 0.14, Δ*F*(8,153) = 3.21, *p* < 0.01] and accounts for almost all of the explained variance.

In the final model, only stress (β = 0.25, *p* < 0.01), smoking (β = 0.26, *p* < 0.01) and alcohol consumption (β = -0.18, *p* = 0.04) are significant predictors of the disinhibition score. The B-values indicate that for each unit that stress (range: 1–4) and smoking increased, a rise in disinhibition of 2.7 and 0.9 points (respectively) is expected. For each alcohol consumption unit [range: 0 (none)–6 (every day)], a decrease of 0.6 points would be expected. The effect size of violent video game exposure during the past week increased from the second model (β = 0.09, *p* = 0.26) to the final model (β = 0.16, *p* = 0.08). Therefore, violent video game exposure significantly predicts higher disinhibition scores [β = 0.16, *p* (1-tailed) = 0.04]. The B-value of 1.00 suggests that a difference in exposure from none to > 10 h in the last week leads to an increase of 12.5% (5 points) on the disinhibition scale. Non-violent video gaming (β = 0.03, *p* = 0.69), on the other hand, does not predict disinhibition scores.

#### Long-Term Versus Recent Video Game Exposure

Lastly, an attempt to disentangle long-term and recent VG exposure was made by adding years of video game exposure to the regressions described above. However, these results have to be viewed with extra caution as due to the non-gamers these factors share substantial common variance with each other. Interestingly, for the interpersonal-affective deficits and overall trait score, recent violent video game exposure remained a significant predictor (interpersonal-affective: β = 0.19, *p* = 0.02; overall traits: β = 0.21, *p* = 0.02), whereas life-time exposure duration did not turn significant (all |β|≤ 0.14, all *p* ≤ 0.11). For disinhibition, however, recent violent video game exposure turned insignificant (β = 0.13, *p* = 0.15), while the added life-time exposure was a significant predictor for relatively higher disinhibition scores [β = 0.16, *p* (1-tailed) = 0.04].

## Discussion

This study set out to investigate the existence of a link between violent video game exposure and adverse behavior traits which are marked by two domains, namely the interpersonal-affective deficits and disinhibition. The results suggest that there indeed is a link between violent video game exposure and psychopathic traits. The link was stronger for recent video game exposure and was stronger associated with the interpersonal-affective deficits. Disinhibition traits, on the other hand, were better predicted by stress and smoking behavior, but were also predicted by violent video game exposure.

### Lifetime Violent Video Game Exposure and Adverse Behavior Traits

The previously hypothesized link between ongoing long-term violent video gaming and the expression of a collection of traits associated with adverse behavior including interpersonal affective deficits as well as disinhibition is supported by this study. For all trait expression scores, significant differences of medium effect sizes were detected for differing (violent) video game exposure. Planned simple contrasts revealed that especially ongoing violent video game exposure was associated with significantly higher interpersonal-affective deficits as well as with increased disinhibition scores. Even though the effect sizes for these simple contrasts tended to be mostly small, these findings are congruent with previous studies investigating the link of violent video gaming with specific traits such as lack of empathy (Anderson et al., 2010; Fraser et al., 2012), impaired morality (Hartmann and Vorderer, 2010; Bajovic, 2013; Gabbiadini et al., 2013), and aggressiveness (Willoughby et al., 2012; DeLisi et al., 2013; Calvert et al., 2017); and is also in line with the bidirectional link between sadism and violent video game exposure, which was illustrated by a recent study of Greitemeyer and Sagioglou (2017). Therefore, evidence – suggesting that violent video gaming can be linked to antisocial traits – is accumulating.

For the overall collection of traits and more specifically for the disinhibition score, a significant difference was found between SVVG and no video game use. For the overall score and the interpersonal-affective deficit score, the effect sizes for the difference with the stopped violent video game exposure were smaller than for the ongoing exposure comparison. These findings may point toward the notion that effects of violent video games fade out after stopped exposure (Anderson et al., 2010; Gabbiadini et al., 2012), and that especially ongoing and repeated exposure may be responsible for larger differences in negative behavioral outcomes – particularly related to interpersonal-affective deficits (Anderson et al., 2010; Willoughby et al., 2012). Nevertheless, the present analyses are purely correlational, therefore, a causal relationship cannot be assumed. The directionality of this effect is uncertain. Therefore, it cannot be excluded that maybe individuals with high psychopathic trait disposition are more prone to use violent video games and less likely to stop violent video games.

### Recent Violent Video Game Exposure and Adverse Behavior Traits

When looking at the intensity of violent video game exposure over the past week specifically, the findings were consistent with previous studies indicating that males tend to have higher adverse trait scores than females (Neumann and Hare, 2008; Coid et al., 2009) and that exposure to violence is a risk factor for higher adverse trait development (Krahé and Möller, 2010; DeLisi et al., 2013). Here, violent video game as well as violent movie exposure were significant predictors especially for self-reported interpersonal-affective deficits and overall adverse behavior traits. Interestingly, only for interpersonal-affective deficits gender was a significant predictor. Therefore, being male was indicative of higher interpersonal-affective deficits, whereas no gender-specific effect was found for the disinhibition traits.

Even though the predictive power of the intensity of violent video game exposure was not as high as for the other adverse traits, it was also a significant predictor for disinhibition traits, considering that with increased exposure a higher disinhibition score was expected. This finding is congruent with previous studies which linked violent video gaming to increased aggressiveness (Wei, 2007; DeLisi et al., 2013); as disinhibition traits are believed to be the gateway for antisocial behavior, such as aggression, due to the poor behavioral control associated with these traits (Gabbiadini et al., 2013). Therefore, violent video game exposure seems to be linked to both domains of traits predictive of aggressive behavior, as it relates to both, the emotional aspect of aggression – represented by higher interpersonal affective deficits such as lack of empathy and callousness –, as well as to an decreased behavioral control/increased impulsiveness as captured by the higher disinhibition scores. However, this influence on disinhibition only persisted as long as life-time exposure duration was not added to the analyses. General video game exposure duration in years seems to predict disinhibition traits better than recent violent video game exposure. Since the effect sizes for other lifestyle factors were also relatively substantial especially in the disinhibition model, it is likely that the co-occurrence of these different factors increases the risk of aggressive behavior further.

### Lifestyle Factors and Adverse Behavior Traits

The notion by Hasan et al. (2013) that stress caused by violent video games is the main component which causes changes in trait expression could not be supported by our data. Non-violent video game exposure did not show any meaningful effect, even though many non-violent video games have competitive components which are thought to also cause stress in their users (Anderson and Carnagey, 2009; Adachi and Willoughby, 2011). Nevertheless, stress – independent of video gaming – was found to be a significant factor predicting disinhibition which is in line with Hasan et al.’s (2013) argument that stress may be linked to increased aggression as it may affect impulse and self-control negatively (Duckworth et al., 2013); and this decreased control of behavior may in turn lead to more aggressive behavior. Furthermore, alcohol consumption showed a meaningful relation to interpersonal-affective deficits and smoking to disinhibition and overall traits. It could be argued for the later that this health risk behavior may be linked to stress (Todd, 2004; Keyes et al., 2012), or that it may support the notion that addictive behaviors like smoking, as is suggested for violent video gaming (Grüsser et al., 2007), in general relate to disinhibition (Flory and Manuck, 2009). Nevertheless, even with these potential confounders violent video game exposure remained an influential factor. Therefore, a link of violent video game exposure with interpersonal-affective deficits in particular, but also with disinhibition, seems to exist.

### Limitations and Future Research

Even though this empirical study has multiple strengths such as a relatively large sample, validated measures for the main constructs and elaborate statistical testing including assumption testing, there are also several limitations that have to be mentioned.

First, convenience sampling was used to collect the data. The sample consisted predominantly (65%) of female participants. As this percentage deviates from the percentage of female gamers, gender was added as a covariate in our analysis. This showed that gender explained about 6.5 % of the variance in all adverse behavior traits. The high percentage of female students is representative of the populations of many college populations but may not represent the population of gamers very well. Therefore, the external validity of the findings is limited. It is interesting to note, however, that the number of female gamers is rising (McDaniel, 2016). Expanding our knowledge on the female group of gamers is thus important. Furthermore, as a self-report measure was administered, a response bias – due to the subjective nature of questionnaires – is possible. However, effect sizes and outcomes are comparable to results of the previously mentioned studies. Furthermore, to ensure that games were consistently identified as either violent or non-violent, each mentioned game was rated by experienced gamers for their content of violence and these results were cross-checked with the self-classification of the participants. To avoid such biases, future studies should employ more objective measures of video game use (e.g., inducing exposure during the course of a study in participants with comparable pre-exposure levels, longitudinal designs starting with young children using parental reports etc.) to move away from data purely relying on self-report. Furthermore, individuals of different nationalities filled in the questionnaire, but it was not examined whether potential differences may be due to different cultural backgrounds. However, since all participants were of Westernized origin, no crucial differences in violent video game use and especially psychopathic traits are expected due to a generally underlying individualistic culture.

Furthermore, another drawback of the study is that participants’ exposure did vary among but also within video-game exposure groups. Therefore, the participants within a group (with video game use) may not be homogenous regarding the intensity of exposure they experienced. Additionally, there might also be a between group difference in intensity of video game exposure. More specifically, participants who use violent video games tend to spend more time with video gaming than participants of the other video game exposure groups. Therefore, it is unclear which influence the varying frequency of exposure had on the present findings. In future studies, a more stringent control for exposure frequency is needed. When considering the exposure frequency, especially in the prior week of recording, of all video game groups, the degree of exposure seems to be rather low [1–3 h in past week compared to the 15 h stated by Gentile (2009)] relative to heavy users of video games. Therefore, the mostly small to medium effect sizes found in this study, may not apply to video game users with an increased frequency of use. It may be expected that the found differences in traits leading to adverse behavior may be pronounced as frequency and duration of exposure increases. These differences in psychopathic trait expression accompanied by violent video game exposure may ultimately turn out to be substantial enough to influence social interactions and relationships in real life. However, it is important to mention that even though social interactions could be negatively impacted by violent video game use, the effect sizes do not support claims by some public media reports that violent video games could be the primary reason for extreme violence, as in mass shootings for example.

Furthermore, even though previous studies have found a causal relationship between violent video gaming and changes in adverse behavior tendencies such as aggression, lack of empathy and immorality, no claims of directionality can be made from the present findings due to the correlational design of this study. Since personality traits, like the traits assessed in this study, are relatively stable and latent, these traits may determine violent video game use and not necessarily the other way round. Alternatively, as suggested by Greitemeyer and Sagioglou (2017), this link between personality traits and tendency to play video games may be bidirectional, meaning that individuals with higher adverse behavior traits may be more likely to play video games and the consequent increased exposure could in turn affect expression of these traits. In this study, some insight into directionality was provided by including a group of participants that have SVVG. However, the possibility that adverse behavior traits could also determine whether people stop violent video game use at a given time point cannot be excluded. Therefore, to investigate the causality of a possible effect of psychopathic trait expression, studies with more elaborate (e.g., longitudinal approaches), well-controlled experimental designs are required in the future to disentangle potential bidirectional effects.

Ideally, future studies start to conceptualize these adverse behavior traits on different response levels (i.e., subjective report, neurophysiology and behavioral measures). Next to employing questionnaires, experimental paradigms measuring different aspects of adverse behavior like empathic abilities, impulsivity/stress and aggression could be implemented while neurophysiological measures, such as brain response [e.g., functional magnetic resonance imaging (fMRI) or near infrared spectroscopy (NIRS)] or physiological parameters (e.g., heart rate variability, skin conductance or pupillometry), are recorded. The combination of subjective and physiological measures could then help to provide a better and possibly more complete measure of adverse behavior traits. For future cross-sectional studies, an introduction of four groups (two similarly experienced groups and two groups with no previous experience, who either play a violent or a non-violent video game during the experiment) may be useful to disentangle short-term effects from possible long-term exposure consequences on adverse behavior traits. Furthermore, future studies should also be able to provide more information about the directionality of the effects between violent video game exposure and adverse behavior traits. Here a well-controlled longitudinal study implementing objective and subjective measures of adverse behavior traits as well as an objective measure (i.e., software tracking gaming behavior on participants’ computers) recording the time spent on (violent) video games before first time exposure to such games would be most promising.

## Conclusion

Overall, there seems to be a link between self-reported violent video game exposure and differences in psychopathic trait expression – especially in interpersonal-affective deficits. Even though disinhibition problems were also found to be increased in (violent) video game groups, other stress-related factors seemed to have a higher predictive power than violent video game exposure. It might be unlikely that generally unburdened violent video gamers turn into real-life mass-shooters as often suggested by the public media, but violent video game exposure might in fact impact interpersonal-affective competencies in such a way that social interaction/contacts and relationships may be negatively affected. Therefore, considering the increasing and extensive use of violent video games, it is of utmost importance to continue pursuing the investigation of the potential impact of violent video games on their consumers’ personality traits as well as expressed behaviors.

## Ethics Statement

This study was carried out in accordance with the recommendations of the internal ethics committee of UCR (University College Roosevelt) with informed consent from all subjects. All subjects gave informed consent in accordance with the Declaration of Helsinki. The protocol was approved by the internal ethics committee of UCR. Since this study only included an online questionnaire no written informed consent was obtained. However, all participants were instructed that they should only proceed with the questionnaire, if they feel sufficiently informed and agree with the information given. Furthermore, they were informed that they could anytime withdraw from the study and that their participation is anonymous.

## Author Contributions

A-CSK designed the online-questionnaire battery and collected the data, carried out the statistical analyses, wrote the initial draft of the manuscript, and reviewed/revised the manuscript thereafter. GA contributed to the design of the study, supervised the study’s development and data acquisition, and reviewed and edited the manuscript. BD provided the advice for the data analysis, contributed to/supervised the writing of the manuscript, and reviewed and edited the manuscript.

## Conflict of Interest Statement

The authors declare that the research was conducted in the absence of any commercial or financial relationships that could be construed as a potential conflict of interest.

## Footnotes

1 Averaged total scores of the scales were used. The homogeneity assumption was not met for the interpersonal-affective total (Levene’s test, *p* < 0.05). However, when using log-transformed or *z*-standardized data, which conformed to this assumption, ANCOVA results did not change. Therefore, the original score was kept in the analysis.

## Abbreviations

NVG: no regular video game use
NVVG: non-violent video game use only
SVVG: stopped violent video game use
VVG: ongoing violent video game use

## References

[B1] Adachi P. J. C. Willoughby T. (2011). The effect of video game competition and violence on aggressive behavior: which characteristic has the greatest influence? 1 259–274. 10.1037/a0024908

[B2] Anderson C. A. (2004). An update on the effects of playing violent video games. 27 113–122. 10.1016/j.adolescence.2003.10.009 15013264

[B3] Anderson C. A. Bushman B. J. (2001). Effects of violent games on aggressive behavior, aggressive cognition, aggressive affect, physiological arousal, and prosocial behavior: a meta-analytic review of the scientific literature. 12 353–359. 10.1111/1467-9280.00366 11554666

[B4] Anderson C. A. Carnagey N. L. (2009). Causal effects of violent sports video games on aggression: is it competitiveness or violent content? 45 731–739. 10.1016/j.jesp.2009.04.019

[B5] Anderson C. A. Dill K. E. (2000). Video games and aggressive thoughts, feelings, and behavior in the laboratory and in life. 78 772–790. 10.1037/0022-3514.78.4.772 10794380

[B6] Anderson C. A. Shibuya A. Ihori N. Swing E. L. Bushman B. J. Sakamoto A. (2010). Violent video game effects on aggression, empathy, and prosocial behavior in Eastern and Western countries: a meta-analytic review. 136 151–173. 10.1037/a0018251 20192553

[B7] Bajovic M. (2013). Violent video gaming and moral reasoning in adolescents: is there an association? 50 177–191. 10.1080/09523987.2013.836367

[B8] Bavelier D. Green C. S. Pouget A. Schrater P. (2012). Brain plasticity through the life span: learning to learn and action video games. 35 391–416. 10.1146/annurev-neuro-060909-152832 22715883

[B9] Calvert S. L. Appelbaum M. Dodge K. A. Graham S. Nagayama Hall G. C. Hamby S. (2017). The American Psychological Association Task Force assessment of violent video games: science in the service of public interest. 72 126–143. 10.1037/a0040413 28221065

[B10] Centers for Disease Control and Prevention (2013). Available at: https://www.cdc.gov/healthyyouth/data/yrbs/questionnaires.htm [accessed June 01 2017].

[B11] Cima M. Tonnaer F. Hauser M. D. (2010). Psychopaths know right from wrong but don’t care. 5 59–67. 10.1093/scan/nsp PMC2840845 20053752

[B12] Coid J. Yang M. Ullrich S. Roberts A. Hare R. D. (2009). Prevalence and correlates of psychopathic traits in the household population of Great Britain. 32 65–73. 10.1016/j.ijlp.2009.01.002 19243821

[B13] Colzato L. S. Van Leeuwen P. J. A. Van Den Wildenberg W. P. M. Hommel B. (2010). DOOM’d to switch: superior cognitive flexibility in players of first person shooter games. 1:8. 10.3389/fpsyg.2010.00008 21833191 PMC3153740

[B14] DeLisi M. Vaughn M. G. Gentile D. A. Anderson C. A. Shook J. J. (2013). Violent video games. delinquency, and youth violence: new evidence. 11 132–142. 10.1177/1541204012460874

[B15] Doyle K. L. El-Nokaly M. Hilton M. L. Prickel D. L. Saud A. Schaiper D. R. (2014). Available at: http://www.google.com/patents/WO2003084402A1?cl=en [accessed April 08 2014].

[B16] Duckworth A. L. Kim B. Tsukayama E. (2013). Life stress impairs self-control in early adolescence. 2013:608. 10.3389/fpsyg.2012.00608 23443890 PMC3581033

[B17] Ferguson C. J. (2007). Evidence for publication bias in video game violence effects literature: a meta-analytic review. 12 470–482. 10.1016/j.avb.2007.01.001

[B18] Flory J. D. Manuck S. B. (2009). Impulsiveness and cigarette smoking. 71 431–437. 10.1097/PSY.0b013e3181988c2d 19251874 PMC2713002

[B19] Fraser A. M. Padilla-Walker L. M. Coyne S. M. Nelson L. J. Stockdale L. A. (2012). Associations between violent video gaming, empathic concern, and prosocial behavior toward strangers, friends, and family members. 41 636–649. 10.1007/s10964-012-9742-2 22302216

[B20] Funk J. B. Bechtoldt-Baldacci H. Pasold T. Baumgardener J. (2004). Violence exposure in real-life, video games, television, movies, and the Internet: is there desensitization? 27 23–39. 10.1016/j.adolescence.2003.10.005 15013258

[B21] Gabbiadini A. Andrighetto L. Volpato C. (2012). Brief report: does exposure to violent video games increase moral disengagement among adolescents? 35 1403–1406. 10.1016/j.adolescence.2012.06.001 22766175

[B22] Gabbiadini A. Riva P. Andrighetto L. Volpato C. Bushman B. J. (2013). Interactive effect of moral disengagement and violent video games on self-control, cheating, and aggression. 4 451–458. 10.1177/1948550613509286

[B23] Gentile D. A. (2009). Pathological video-game use among youth ages 8 to 18: a national study. 20 594–602. 10.1111/j.1467-9280.2009.02340.x 19476590

[B24] Gentile D. A. Lynch P. L. Linder J. R. Walsh D. A. (2004). The effects of violent video game habits on adolescent hostility, aggressive behaviors, and school performance. 27 5–22. 10.1016/j.adolescence.2003.10.002 15013257

[B25] Green C. S. Bavelier D. (2003). Action video game modifies visual selective attention. 423 534–537. 10.1038/nature01647 12774121

[B26] Green C. S. Pouget A. Bavelier D. (2010). Improved probabilistic inference as a general learning mechanism with action video games. 20 1573–1579. 10.1016/j.cub.2010.07.040 20833324 PMC2956114

[B27] Greitemeyer T. Mügge D. O. (2014). Video games do affect social outcomes: a meta-analytic review of the effects of violent and prosocial video game play. 40 578–589. 10.1177/0146167213520459 24458215

[B28] Greitemeyer T. Sagioglou C. (2017). The longitudinal relationship between everyday sadism and the amount of violent video game play. 104 238–242. 10.1016/j.paid.2016.08.021

[B29] Grüsser S. M. Thalemann R. Griffiths M. D. (2007). Excessive computer game playing: evidence for addiction and aggression? 10 290–292. 10.1089/cpb.2006.9956 17474848

[B30] Hare R. D. (2003). , 2nd Edn. Canada, ON: Multi-Health Systems.

[B31] Hartmann T. Vorderer P. (2010). It’s okay to shoot a character: moral disengagement in violent video games. 60 94–119. 10.1111/j.1460-2466.2009.01459.x

[B32] Hasan Y. Bègue L. Bushman B. (2013). Violent video games stress people out and make them more aggressive. 39 64–70. 10.1002/ab.21454 23097053

[B33] Huberty C. J. Morris J. D. (1989). Multivariate analysis versus multiple univariate analyses. 105 302–308. 10.1037/0033-2909.105.2.302

[B34] Keyes K. M. Hatzenbuehler M. L. Grant B. F. Hasin D. S. (2012). Stress and alcohol: epidemiologic evidence. 34 391–400. 10.35946/arcr.v34.4.03 PMC3797525 23584105

[B35] Klimmt C. Schmid H. Nosper A. Hartmann T. Vorderer P. (2006). How players manage moral concerns to make video game violence enjoyable. 31 309–328. 10.1515/COMMUN.2006.020

[B36] Krahé B. Möller I. (2010). Longitudinal effects of media violence on aggression and empathy among German adolescents. 31 401–409. 10.1016/j.appdev.2010.07.003

[B37] Levenson M. Kiehl K. Fitzpatrick C. (1995). Assessing psychopathic attributes in a noninstitutionalized population. 68 151–158. 10.1037/0022-3514.68.1.151 7861311

[B38] Lynam D. R. Whiteside S. Jones S. (1999). Self-reported psychopathy: a validation study. 73 110–132. 10.1207/S15327752JPA730108 10497804

[B39] McDaniel M. A. (2016). Available at: https://aquila.usm.edu/honors_theses/427

[B40] Möller I. Krahé B. (2009). Exposure to violent video games and aggression in German adolescents: a longitudinal analysis. 35 75–89. 10.1002/ab.20290 19016226

[B41] Neumann C. S. Hare R. D. (2008). Psychopathic traits in a large community sample: links to violence, alcohol use, and intelligence. 76 893–899. 10.1037/0022-006X.76.5.893 18837606

[B42] Parkes A. Sweeting H. Wight D. Henderson M. (2013). Do television and electronic games predict children’s psychosocial adjustment? Longitudinal research using the UK Millennium Cohort Study. 98 341–348. 10.1136/archdischild-2011-301508 23529828 PMC3625829

[B43] Saleem M. Anderson C. A. Gentile D. A. (2012). Effects of prosocial, neutral, and violent video games on college students’ affect. 38 263–271. 10.1002/ab.21427 22549724

[B44] Seara-Cardoso A. Craig N. Roiser J. McCrory E. Viding E. (2012). Investigating associations between empathy, morality and psychopathic personality traits in the general population. 52 67–71. 10.1016/j.paid.2011.08.029

[B45] Siyez D. M. Baran B. (2017). Determining reactive and proactive aggression and empathy levels of middle school students regarding their video game preferences. 72 286–295. 10.1016/j.chb.2017.03.006

[B46] Szycik G. R. Mohammadi B. Münte T. F. te Wildt B. T. (2017). Lack of evidence that neural empathic responses are blunted in excessive users of violent video games: an fMRI study. 8:174. 10.3389/fpsyg.2017.00174 28337156 PMC5341328

[B47] Todd M. (2004). Daily processes in stress and smoking: effects of negative events, nicotine dependence, and gender. 18 31–39. 10.1037/0893-164X.18.1.31 15008683

[B48] Vallett D. B. Lamb R. L. Annetta L. A. (2013). The gorilla in the room: the impacts of video-game play on visual attention. 29 2183–2187. 10.1016/j.chb.2013.05.001

[B49] Wallenius M. Punamäki R. L. (2008). Digital game violence and direct aggression in adolescence: a longitudinal study of the roles of sex, age, and parent–child communication. 29 286–294. 10.1016/j.appdev.2008.04.010 27519031

[B50] Wei R. (2007). Effects of playing violent video games on Chinese adolescents’ pro-violence attitudes, attitudes toward others, and aggressive behavior. 10 371–380. 10.1089/cpb.2006.9942 17594261

[B51] Willoughby T. Adachi P. J. C. Good M. (2012). A longitudinal study of the association between violent video game play and aggression among adolescents. 48 1044–1057. 10.1037/a0026046 22040315

